# Repetitive Myocardial Infarctions Secondary to Delirium Tremens

**DOI:** 10.1155/2014/638493

**Published:** 2014-08-12

**Authors:** David Schwartzberg, Adam Shiroff

**Affiliations:** ^1^Department of Surgery, Monmouth Medical Center, Long Branch 300 2nd Avenue, Long Branch, NJ 07760, USA; ^2^Department of Trauma & Critical Care Surgery, Jersey Shore University Medical Center, Neptune, NJ 07753, USA

## Abstract

Delirium tremens develops in a minority of patients undergoing acute alcohol withdrawal; however, that minority is vulnerable to significant morbidity and mortality. Historically, benzodiazepines are given intravenously to control withdrawal symptoms, although occasionally a more substantial medication is needed to prevent the devastating effects of delirium tremens, that is, propofol. We report a trauma patient who required propofol sedation for delirium tremens that was refractory to benzodiazepine treatment. Extubed prematurely, he suffered a non-ST segment myocardial infarction followed by an ST segment myocardial infarction requiring multiple interventions by cardiology. We hypothesize that his myocardial ischemia was secondary to an increased myocardial oxygen demand that occurred during his stress-induced catecholamine surge during the time he was undertreated for delirium tremens. This advocates for the use of propofol for refractory benzodiazepine treatment of delirium tremens and adds to the literature on the instability patients experience during withdrawal.

## 1. Introduction

Delirium tremens (DTs), the most feared consequence of acute alcohol withdrawal, can lead to hallucinations, delirium, seizures, myocardial infarction, all of the above, or death [1, 2]. Several authors have documented ST changes and electrocardiogram abnormalities associated with ischemia in the setting of alcohol withdrawal [2–4]. We report a non-ST segment elevation myocardial infarction (NSTEMI) as well as an ST segment elevation myocardial infarction (STEMI) in the setting of delirium tremens refractory to treatment with benzodiazepines (BZ) [3, 4]. This case report adds to the scarce literature documenting myocardial ischemia associated with DTs.

## 2. Case Presentation

A 65-year-old male was admitted to the trauma service after a mechanical fall down from five cement stairs. The result was multiple left sided rib fractures and a small lung contusion without loss of consciousness. He was admitted for oxygen therapy and pain control with a patient-controlled-anesthesia unit, along with thiamine and Librium [Valeant Pharmaceuticals, Bridgewater, NJ] once it was evident he had a history of significant alcohol abuse. The patient had no primary care physician so there was no documented past medical history; however, he did not report angina symptoms. Without signs of alcohol withdrawal, he was scheduled for discharge 36 hours after admission. The night before his expected discharge he became tachycardic, hypertensive (systolic to 200 mmHg from baseline of 120 mmHg), and lethargic and was treated by beta-blocker, clonidine patch, nil per os (NPO) and upgraded to the intensive care unit. He was subsequently intubated for lethargy and was started on propofol, fentanyl, and tube feeds. He was stabilized and remained an 11 t on the Glascow Coma Scale and after a successful weaning trial he was extubated less than 24 hours later after intubation.

**Figure 1:**
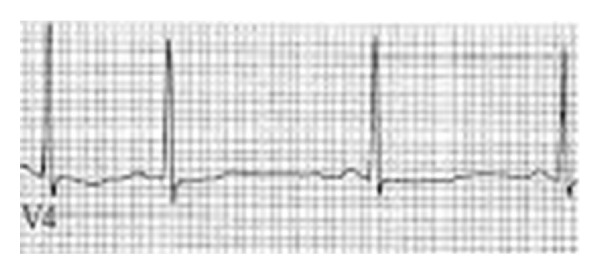
Evidence of NSTEMI, along with elevated troponins; the patient underwent cardiac stent placement.

Once extubated he quickly became tachycardic and hypertensive but responded appropriately. It was decided that he would be treated symptomatically with intravenous BZ even though they proved ineffective previously. Hours later, he underwent rapid sequence intubation after prolonged hypoxia, respiratory distress, and tachycardia. Secondary to his hemodynamic instability, a 2D cardiac echocardiogram was performed which showed a left ventricle ejection fraction of 35%. He went into an episode of ventricular tachycardia as troponin of 22 ng/ml resulted along with electrocardiogram changes diagnosing a non-ST segment myocardial infarction (Figure 1). This was presumed to be because of the increased myocardial oxygen demand associated with a prolonged interval of inadequate DTs treatment. Interventional cardiology took the patient to the catheterization laboratory where angioplasty and drug-eluting stents were placed in his left anterior descending (LAD) and left circumflex artery (LCA) for severe calcification and stenosis. Effient [Eli Lilly and Co., Indianapolis, IN], aspirin, a beta-blocker, and statin were started postprocedurally.

**Figure 2:**
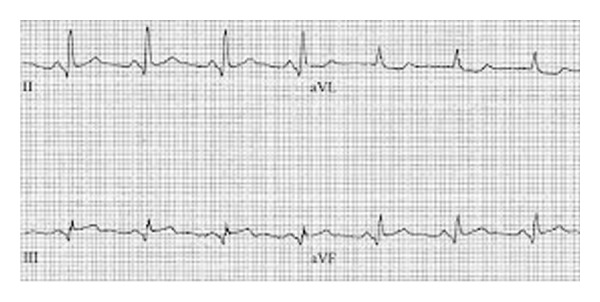
ST segment elevation in lead II, III, and aVF with elevation of troponins; additional stent placed 72 hours s/p LAD & LCA stent placement.

72 hours later, he was persistently oliguric and hypotensive and troponins were elevating (stable at 4 ng/ml then elevating to 32 ng/ml over 12 hours). A repeat electrocardiogram was performed showing an ST segment elevation myocardial infarction in the inferior leads (Figure 2). He was taken back to the catheterization laboratory where his distal right coronary artery underwent angioplasty and stenting for 90% stenosis. He was then transferred to the coronary care unit and started on vasoactive medication to maintain an adequate cardiac output. The dye load encountered during his cardiac procedures combined with periods of a low-flow state resulted in acute tubular necrosis and acute kidney insufficiency; the family refused hemodialysis as the made him do-not-resuscitate (DNR).

Once the patient was stabilized, over the course of a week, his sedation was weaned. It became evident that the patient was not responsive and his pupils were uneven with successful weaning of all sedation/narcotics. A computed tomography of the head was performed showing bilateral cerebral hemispheric infarcts as well as a 20 mm midline shift and partial effacement of the fourth ventricle. Neurosurgery recommended nonoperative care and started the patient on mannitol for cerebral edema, labeling him with a dismal prognosis. He developed Staphlyococcus pneumonia and was started on antibiotics appropriately. Twenty days after admission the patient went into asystole and expired. He never recovered from his myocardial ischemia induced by the catecholamine surge caused by delirium tremens.

## 3. Discussion 

Our delirium tremens patient suffered from both NSTEMI and STEMI, which as a combined insult proved to be unrecoverable. He obviously had underlying cardiac stenosis, evident by his catheterization; however, his oxygen demand was exponentially increased during the brief period of time when he was extubated resulting in myocardial ischemia. Prior to being weaned from propofol, his vital signs were stable and he seemed to be enduring his alcohol withdrawal. This supports literature stating propofol should be used in DTs refractory to treatment with BZ. Although it is unproven, it would seem that his premature cessation of propofol resulted in a cascade of events that caused hemodynamic instability, myocardial ischemia, and multiple cardiac catheterization procedures that likely caused his resulting cerebral infarcts.

Alcohol withdrawal symptoms are present in up to 31% of trauma patients, with studies reporting delirium in 4–15% of all patients with alcohol dependence admitted to the hospital [1]. Prototypical treatment of DTs has been benzodiazepines to control symptoms and avoid the detrimental effects of alcohol withdrawal [1]. The base for treatment is minimizing the effects that chronic alcohol dependency has on neuromodulation and brain receptors. Ethanol affects two key receptors in the brain responsible for depressive or excitatory activity. Inhibitory gamma-aminobutyric acid (GABA) receptors are downregulated because of ethanol's intrinsic depressive effect while the excitatory N-methyl-D-asparate (NMDA) receptors are upregulated to maintain equilibrium [1, 5].

Benzodiazepine therapy is successful in mimicking the inhibitory effects of GABA, which is unopposed due to the absence of ethanol. Unfortunately, BZ treatment does not have any effect on the upregulated NMDA receptors which are unopposed, as this is the mechanism of BZ treatment failure. The upregulated NMDA receptors are directly treated with propofol sedation, which not only mimics GABA-receptors but also blocks the excitatory NMDA-receptor pathway. Because propofol acts on both receptors, it has been promoted to second line treatment and proven as the leading medication to treat patients who are refractory to BZ treatment [6, 7]. Previous studies documented successful avoidance of delirium tremens with the use of propofol since the first reported case in 1997 [8, 9]. The overt drawback of propofol is its high price, intensive care unit monitoring, and intubation (secondary to propofol's respiratory depressive effects), which is why it remains a second line therapy. Recently, a study advocating for the use of dexmedetomidine (Precidex) [Orion Pharmaceuticals, Espoo, Finland] because of its sedative qualities without causing respiratory depression showed promise in the setting for DTs refractory to BZ treatment; however, this idea needs further investigation [8].

Propofol's ability to mediate the hemodynamic instability during alcohol withdrawal is paramount in patients refractory to BZ treatment and should be started promptly when recognized. There is documented variability of QT intervals and ST segment changes associated with withdrawals as well as increases in myocardial oxygen demand secondary to a catecholamine surge [10, 11]. QT variability can lead to re-entry tachycardia and is arrhythmogenic, especially with the electrolyte imbalance most alcoholics possess. Even without a known cardiac history, a Swedish paper showed sympathoadrenergic instability influencing the release of myocardial enzymes and causing ST segment changes. In their study, downsloping ST segment depression was seen in over one-third of their 19-patient accruement [11].

## 4. Conclusion

This is an additional reported case on a very scarcely reported subject documenting myocardial infarction in the setting of delirium tremens. Although cases have shown DTs causing ST segment variability and a propensity towards arrhythmias, our patient seems to have suffered myocardial infarction because of myocardial stress. His myocardial stress was secondary to a catecholamine surge and resulting hemodynamic instability from delirium tremens.
